# Downregulation of MARC2 Promotes Immune Escape and Is Associated With Immunosuppression of Hepatocellular Carcinoma

**DOI:** 10.3389/fgene.2021.790093

**Published:** 2022-01-31

**Authors:** Dehai Wu, Shuhang Liang, Hongrui Guo, Shugeng Zhang, Guangchao Yang, Yubin Yuan, Lianxin Liu

**Affiliations:** ^1^ Department of Hepatic Surgery, Key Laboratory of Hepatosplenic Surgery, Ministry of Education, The First Affiliated Hospital of Harbin Medical University, Harbin, China; ^2^ Department of General Surgery, Heze Municipal Hospital, Heze, China

**Keywords:** N-reductive enzyme system, MARC2, hepatocellular carcinoma, lipid metabolism, immune escape

## Abstract

The N-reductive enzyme system (NRES), composed of MARC1, MARC2, CYB5, and CYB5R, is responsible for the reduction of N-oxygenated compounds and participates in several physiological processes. For example, MARC2 serves as an important prognostic indicator and is downregulated in hepatocellular carcinoma, and the downregulation of MARC2 is critical to the regulation of lipid metabolism and cell cycle progression. However, the role of MARC2 in tumor immune microenvironment modification had not previously been investigated. In this study, we found that downregulation of MARC2 was associated with the differentiation of CD4+T cells into regulatory T cells (Tregs). Furthermore, restoring the expression of MARC2 could increase the expression of HLA-C and B2M via PPARA-related lipid metabolism signaling pathways, which could facilitate tumor antigen presentation to the tumor-infiltrating T cells. Additionally, MARC2 expression negatively correlated with several immune checkpoints. The immune checkpoint burden was generated based on 28 MARC2-related immune checkpoints. Patients with a higher immune checkpoint burden were predicted to have a poorer prognosis and a lower level of activated CD8^+^ T cells. The results showed that expression of the NRES is a prognostic indicator of hepatocellular carcinoma and MARC2 contributes significantly to predict the prognosis. Finally, loss of MARC2 in HCC patients was found to facilitate immune escape and was associated with immunosuppression.

## Introduction

The N-reductive enzyme system (NRES), composed of MARC1, MARC2, CYB5, and CYB5R, is responsible for the reduction of N-oxygenated compounds (Wahl et al., 2010; Havemeyer et al., 2011). These compounds can be small, inorganic or organic molecules, with chemical bonds between nitrogen and oxygen participating in various functions. The NRES plays an important role in the activation of N-hydroxylated prodrugs such as amidoximes and the detoxification of physiological metabolites such as trimethylamine N-oxide (Gruenewald et al., 2008; Schneider et al., 2018). In addition, the NRES could also regulate the L-arginine-dependent biosynthesis of NO and restore the N-hydroxylated nucleobases-which have been shown to be mutagenic for prokaryotic and eukaryotic cells-to the original nucleobases (Khromov-Borisov, 1997; Kotthaus et al., 2011; Rixen et al., 2019). Furthermore, the NRES has been found to be involved in the regulation of lipid metabolism (Neve et al., 2012).

MARC2 is important for N-oxygenation reduction and lipid metabolism (Neve et al., 2012). Only low residual reductive activity was still detectable in MARC2 knockout mice and knockout of MARC2 could affect the energy pathway (Rixen et al., 2019). MARC2 is abundant in the adult liver, omental, and subcutaneous fat, but not in the fetal liver (Neve et al., 2015). Recent studies have shown that MARC2 is associated with tumor progression. In one study, during the transition from normal colon mucosa to adenoma and adenocarcinoma, the expression of MARC2 decreased, indicating that there was a correlation between the loss of MARC2 and colon cancer onset (Mikula et al., 2011). Additionally, our previous study verified that MARC2 expression inhibited the development of hepatocellular carcinoma (HCC) by elevating the expression of p27 (Wu et al., 2020).

In the present study, we found that the NRES is involved in the progression of HCC. The risk score based on the Cox regression analysis implied that the prognostic value of NRES and MARC2 contributed significantly to the prognostic value of the NRES. Our previous study emphasized that MARC2 induces cell cycle arrest in HCC (Wu et al., 2020). In the present study, we investigated the role of MARC2 in tumor immune microenvironment modification and found that it could be used as a marker for response to immunotherapy.

## Materials and Methods

### Acquirement of HCC Data From TCGA Datasets

We obtained TCGA-HCC, MSI MANTIS, MSIsensor, Fraction Genome Altered, Aneuploidy Score, Ragnum hypoxia score, Buffa hypoxia score, and Winter hypoxia score from the TCGA project (https://www.cbioportal.org/).

### Immune Score Calculated Based on the TCGA-HCC Dataset

The immune cell type and tumor mutation burden (TMB) scores were obtained from the open public data platform, Sangerbox (http://sangerbox.com/Index). The cell type identification by estimating relative subsets of RNA transcripts (CIBERSORT) method was used to measure immune cell infiltration in tumor tissues (https://cibersort.stanford.edu/index.php) (Newman et al., 2015; Chen et al., 2018). The data were obtained using the TIMER platform (http://cistrome.dfci.harvard.edu/TIMER/).

### Survival Analysis

The risk score was calculated using the following formula: risk score = β1×1 + β2×2 +… + βixi, where xi is the expression level of each gene, and βi is the risk coefficient of each gene derived from the Cox model (Aguirre-Gamboa et al., 2013). The Kaplan-Meier curve was used to compare the survival rates between different groups, and the log-rank test was used to compare survival between different groups. The threshold was defined as *p* < 0.05.

### Western Blot

Cells were lysed in RIPA buffer containing protease and phosphatase inhibitors. The samples were separated by SDS-PAGE and transferred to polyvinylidene fluoride membranes. After blocking with 5% skim milk at room temperature for 1 h, the membranes were incubated with primary antibodies overnight. Then, the membranes were incubated with the secondary antibodies for 1 h at room temperature, and the bands were detected using an enhanced chemiluminescence kit. Antibodies against MARC2 (ab224097), PPARA (ab126285), HLA-C (ab126722), and B2M (ab75853) were obtained from Abcam.

### Statistical Analysis

R studio and GraphPad Prism 7 were used for the statistical analysis. Statistical analysis for two groups was performed using Student’s t-test. The log-rank test was used for the statistical analysis of survival curves. Linear regression analysis was used to determine the correlation between the two groups.

## Results

### NRES Expression Was Associated With HCC Progression

**FIGURE 1:**
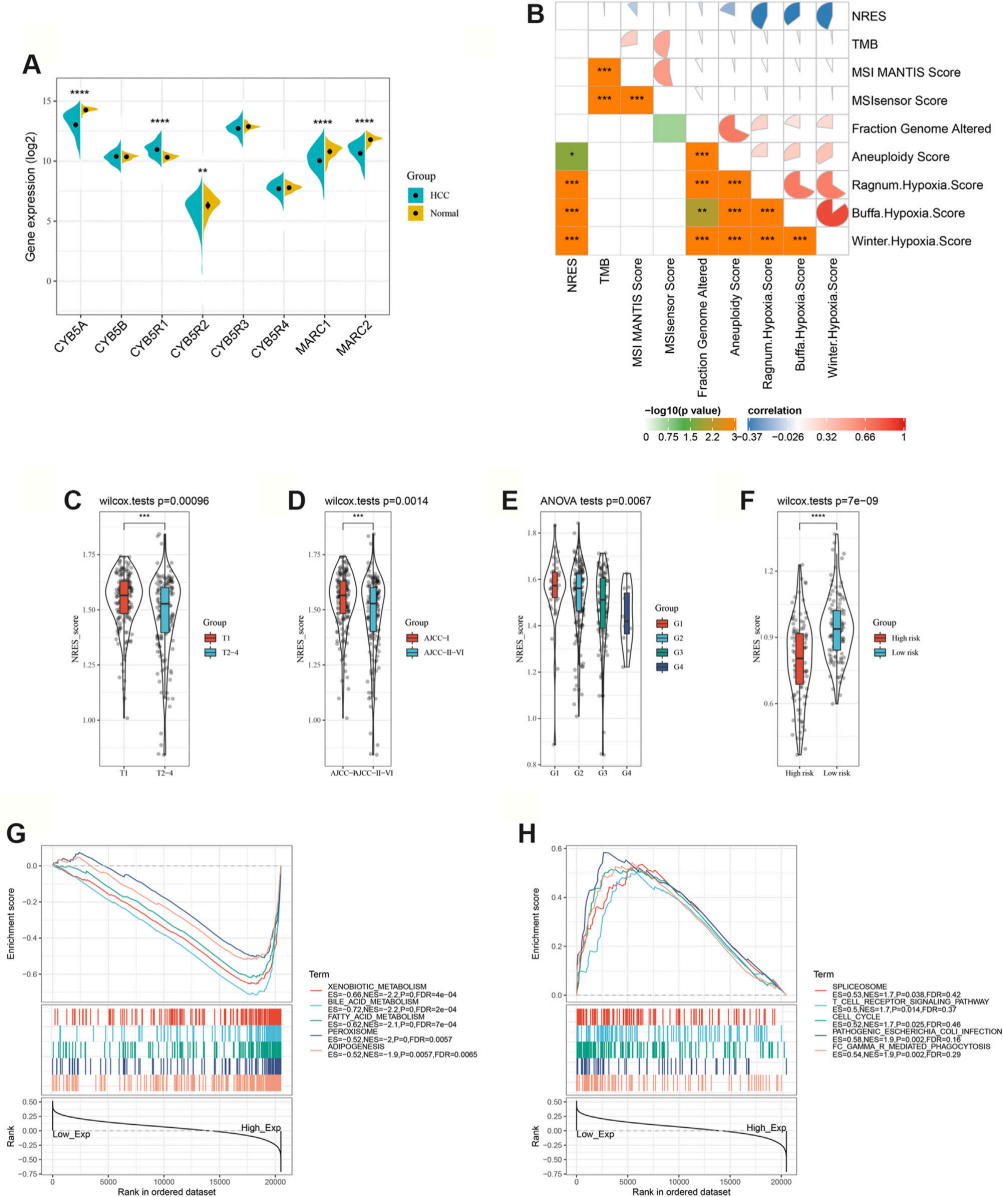
NRES expression was associated with HCC progression. **(A)** Compared the gene expressions of NRES components between normal and HCC tissues. **(B)** The correlation between NRES and different clinical scores. **(C–F)** Compared the gene expressions of NRES components between different groups. Tumor size (T), AJCC stage and tumor pathology grade (G) were obtained from TCGA-LIHC datasets; metastasis risk for HCC was obtained from GEO14520 datasets. **(G–H)** Signaling pathways significantly correlated with NRES. Data was generated by GSEA. The Pearson correlation coefficient was used to determine the correlation. *p<0.05; **p<0.01; ***p<0.001.

The elements of the NRES were dysregulated in HCC patients (Figure 1A). The expression level of the NRES was derived from the ssGSEA (Gene set enrichment analysis) method based on the gene expression of MARC1, MARC2, CYB5A, CYB5B, CYB5R1, CYB5R2, CYB5R3, and CYB5R4. TMB, microsatellite instability (MSI), and hypoxia are biomarkers of immune checkpoint inhibitors. Here, we found that the NRES levels had a significant, negative correlation with hypoxia scores (Figure 1B). Furthermore, patients with lower NRES expression were predicted to have larger tumors, more advanced tumor staging, and a pathological stage (Figures 1C–E). Moreover, a lower level of NRES expression was associated with a higher risk of metastasis (Figure 1F). GSEA showed that the gene sets related to metabolic signaling pathways, such as xenobiotic metabolism, bile acid metabolism, and fatty acid metabolism were enriched in patients with higher levels of NRES expression, while the gene sets related to the immune signaling pathway, such as the T cell receptor signaling pathway and FC gamma R-mediated phagocytosis, were enriched in patients with lower levels of NRES expression (Figures 1G,H). These results revealed that NRES expression participated in the progression of HCC and might be involved in the crosstalk between metabolic reprogramming and tumor immune response.

### NRES Exhibited a Prognostic Value for HCC

**FIGURE 2:**
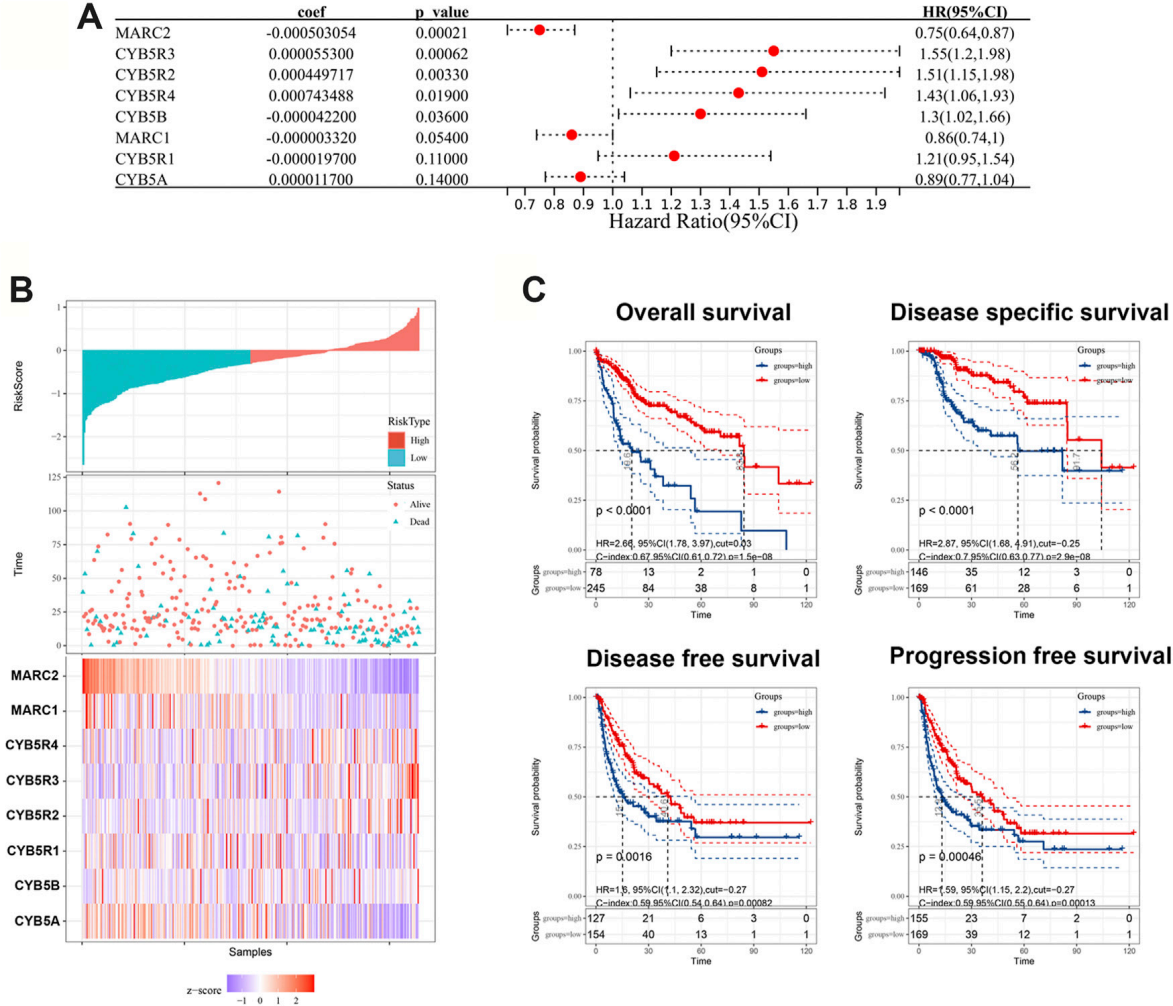
NRES exhibited a prognostic value for HCC. **(A)** Cox regression analysis was performed by R “survival” and “survminer” packages to evaluate the relationship between gene expression and OS. **(B)** The risk scores were created by adding up the product of coefficient of each gene and gene expression. **(C)** The Kaplan-Meier curves for OS, DFS, DFS and PFS; log-rank test was used to compare survival between different groups. The blue curves represented the survival rate of patient with higher level of risk score and the red curves represented the survival rate of patient with lower level of risk score.

To explore the prognostic value of the NRES, the NRES genes were entered into a stepwise multivariate Cox regression analysis. Additionally, univariate Cox regression analysis was performed to evaluate the relationship between gene expression and overall survival (OS) (Figure 2A). MARC2 exhibited a stronger protective effect in patients with HCC than other compounds of NRES (Figure 2A). Then, the risk scores were created by adding the product of the coefficient of each gene and gene expression (Figure 2B). Patients with higher risk scores had shorter OS, disease-specific survival (DSS), disease-free survival (DFS), and progression-free survival (PFS) (Figure 2C). Cox regression analysis showed that the risk score was an independent risk factor for HCC (Table 1). The results implied that the prognostic value of the NRES and MARC2 exhibited a more prominent prognostic value.

**TABLE 1:** Univariate and multivariate analysis. Highlight the significant *p* value.

Overall survival				
	Univariate		Multivariate	
	HR (95%CI)	*p value*	HR (95%CI)	*p value*
Age	1.013 (0.998–1.029)	0.082		
Gender	1.302 (0.883–1.919)	0.183		
AJCC	1.678 (1.366–2.061)	***<0.001***		
Tumor Size	1.652 (1.353–2.017)	***<0.001***		
Lymph node	1.376 (0.903–2.097)	0.138		
Metastasis	1.710 (1.133–2.581)	***0.011***	1.653 (1.029–2.656)	***0.038***
Tumor Grade	1.113 (0.861–1.440)	0.414		
Risk score	2.718 (1.807–4.090)	***<0.001***	2.205 (1.452–3.349)	***<0.001***

### The Expression of MARC2 Was Associated With Tumor Immune Microenvironment Modification and Lipid Metabolism

**FIGURE 3:**
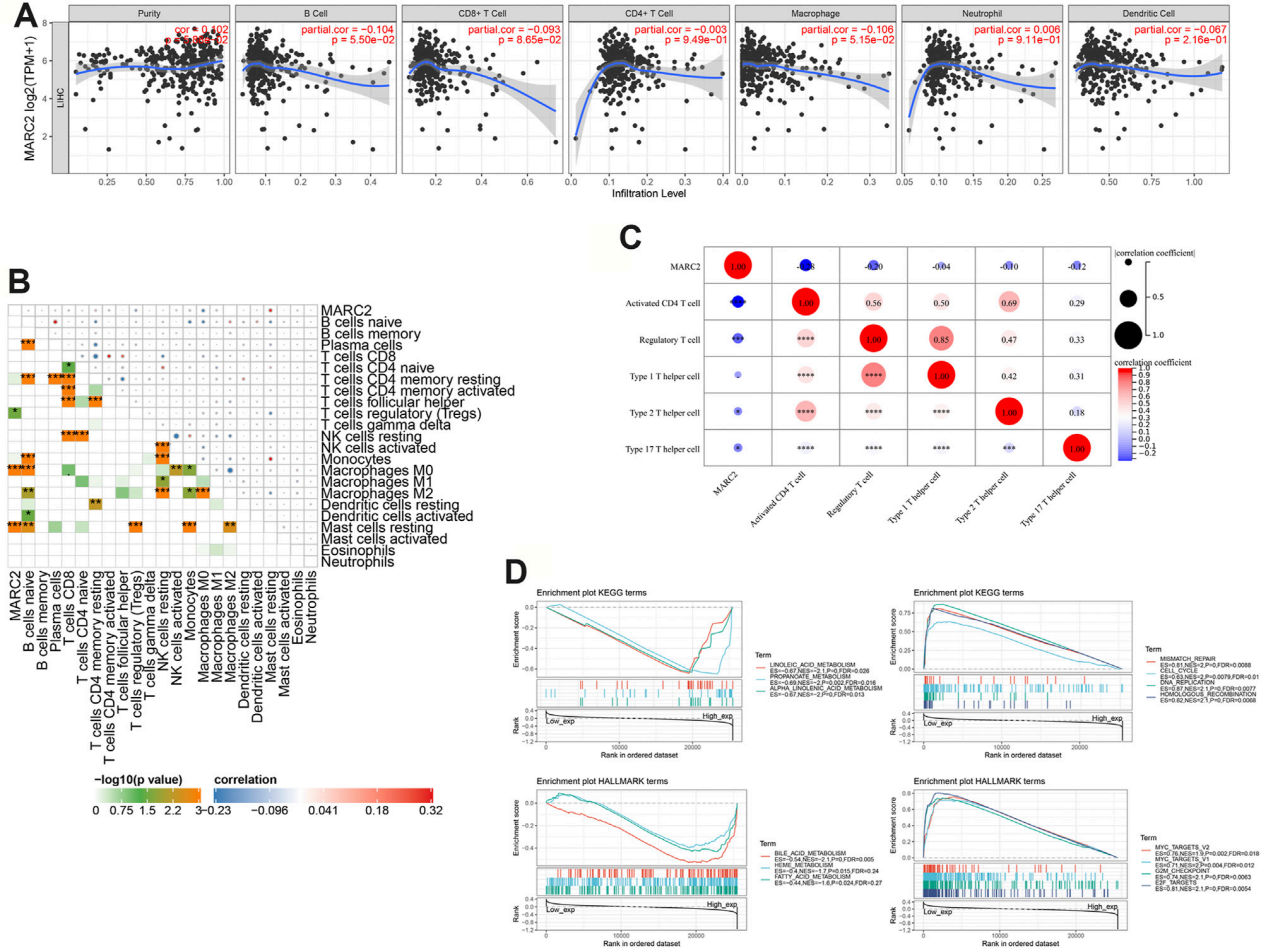
The expression of MARC2 was associated with tumor immune microenvironment modification and lipid metabolism. **(A)** Correlation between MARC2 and tumor infiltrating immune cell. **(B)** The correlation between MARC2 and immune cell type. **(C)** The correlation between MARC2 and CD4+T cell related differentiation subgroups. **(D)** Signaling pathways significantly correlated with MARC2. Data was generated by GSEA. The Pearson correlation coefficient was used to determine the correlation. *p<0.05; **p<0.01; ***p<0.001; ***p<0.0001.

A previous study showed that MARC2 was downregulated in HCC and served as a tumor suppressor by regulating the expression of p27 (Wu et al., 2020). However, the role of MARC2 in the tumor immune microenvironment has not yet been investigated. The immune cells infiltrating HCC were analyzed using a web server for comprehensive analysis of tumor-infiltrating immune cells called TIMER (https://cistrome.shinyapps.io/timer/). The results showed that gene expression of MARC2 had no relationship with tumor-infiltrating immune cells in HCC patients (Figure 3A). Cox regression analysis revealed that MARC2 was an independent risk factor for HCC after adjusting for immune cell infiltration (Table 2). Then, the immune cell types were adopted from CIBERSORT. We found that expression of MARC2 was positively correlated with resting mast cells and negatively correlated with M0 macrophages, and Tregs, a subset of CD4+T cells (Figure 3B). Moreover, MARC2 was significantly negatively correlated with activation of CD4+T cells and slightly negatively associated with Th1, Th2, and Th17 subsets, indicating that MARC2 downregulation in HCC patients was correlated with the differentiation of CD4+T cells into Tregs (Figure 3C). GSEA analysis revealed that lipid metabolism-related gene sets were enriched in patients with high levels of MARC2, and gene sets related to the cell cycle process were enriched in patients with lower levels of MARC2 (Figure 3D).

**TABLE 2:** Cox regression analysis.

	Coef	HR	95%CI_l	95%CI_u	p.value	Sig
B_cell	–7.788	0.000	0.000	0.493	0.031	*
CD8_Tcell	–9.108	0.000	0.000	0.014	0.000	***
CD4_Tcell	–4.310	0.013	0.000	6.032	0.167	
Macrophage	4.236	69.148	0.513	9,329.480	0.090	·
Neutrophil	4.493	89.402	0.002	4836997.582	0.419	
Dendritic	5.800	330.135	10.985	9,921.493	0.001	**
MARC2	–0.351	0.704	0.594	0.834	0.000	***

### MARC2 Facilitated the Tumor Antigen Presentation via PPARA Signaling Pathway

**FIGURE 4:**
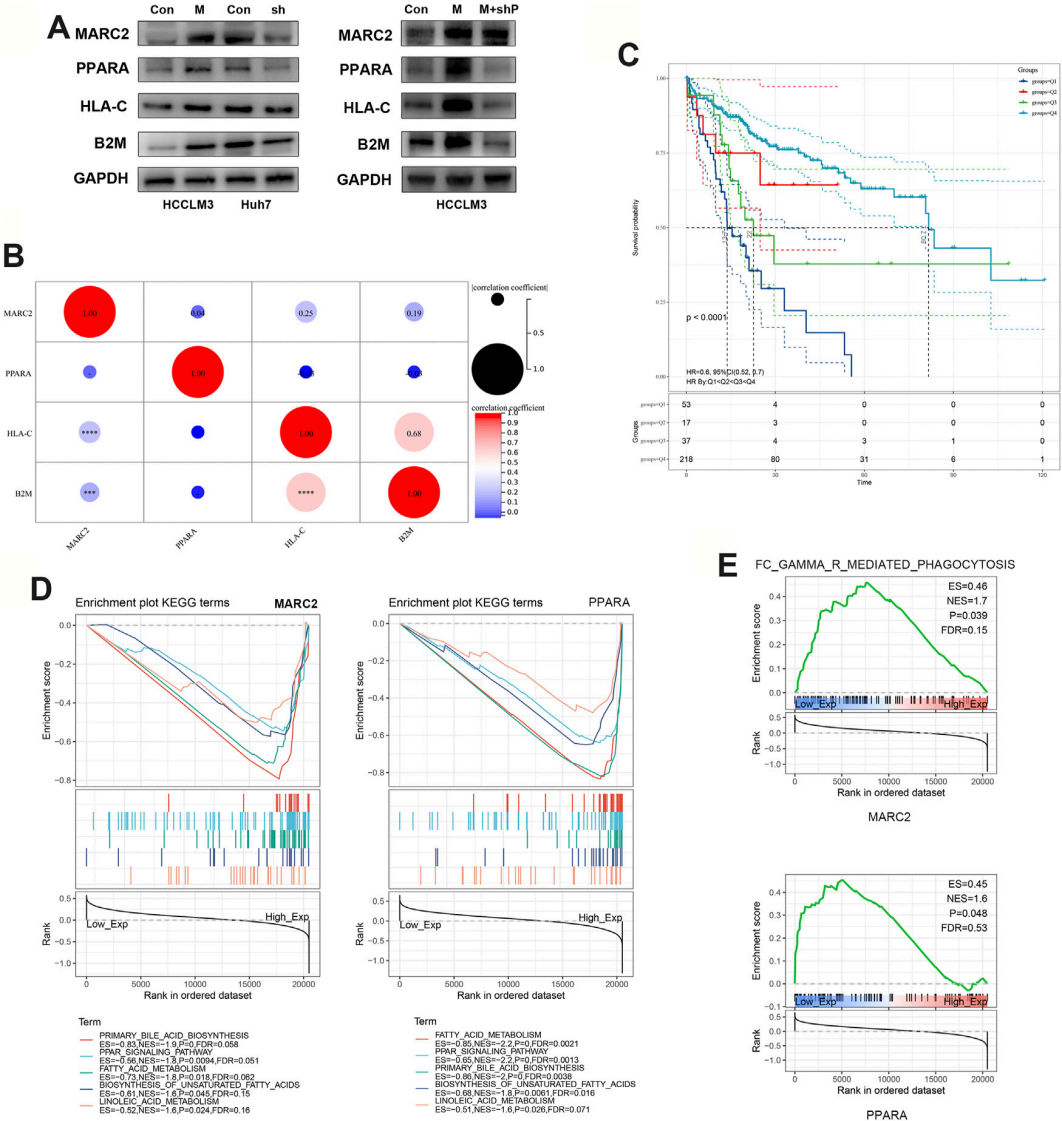
MARC2 facilitated the tumor antigen presentation via PPARA signaling pathway. **(A)** Western blot was used to measure the protein expression of MARC2, PPARA, HLA-C, and B2M in the indicated cell lines. Control: HCCLM3 transfected with control plasmid; MARC2: HCCLM3 transfected with MARC2 overexpression plasmid; sh: HCCLM3 transfected with PPARA knockdown plasmid. **(B)** The correlation between MARC2 and PPARA, HLA-C and B2M. **(C)** The prognostic value of the combination of MARC2 and CD8+T cell was determined by Kaplan-Meier curves. Q1: MARC2 low and CD8+T cell low; Q2: MARC2 low and CD8+T cell high; Q3: MARC2 high and CD8+T cell low; Q4: MARC2 high and CD8+T cell high. **(D–E)** Signaling pathways related with MARC2 and PPARA. The Pearson correlation coefficient was used to determine the correlation. ***p<0.001; ***p<0.0001.

We found that restoring the expression of MARC2 increased the expression of PPARA and major histocompatibility complex-related (MHC) protein 1, HLA-C, as well as B2M (Figure 4A). Decreased PPARA expression attenuated the effect of MARC2 on HLA-C and B2M, indicating that MARC2 regulates the expression of HLA-C and B2M via PPARA (Figure 4A). Expression of MARC2 also showed a moderate correlation with expression of HLA-C and B2M in the TCGA-HCC cohort (Figure 4B). HLA-C and B2M are required to present tumor neoantigens to cytotoxic CD8+T cells. The lack of HLA-C and B2M limits the activation of tumor-infiltrating T cells (Tran et al., 2015). The prognostic value of the combination of MARC2 and tumor-infiltrating CD8+T cells was determined using the Kaplan-Meier curve. The results showed that patients with higher levels of MARC2 and tumor-infiltrating CD8+T cells were predicted to have a longer survival time (Q4), confirming that MARC2 is involved in the antitumor immune process. GSEA results demonstrated that both MARC2 and PPARA were associated with the same lipid metabolism pathways and immune signaling pathway, implying that PPARA-related lipid metabolism is a potential mechanism of MARC2-dependent immune modification (Figures 4D,E).

### There Was a Correlation Between MARC2-Related Lipid Metabolism and Immune Modification

**FIGURE 5:**
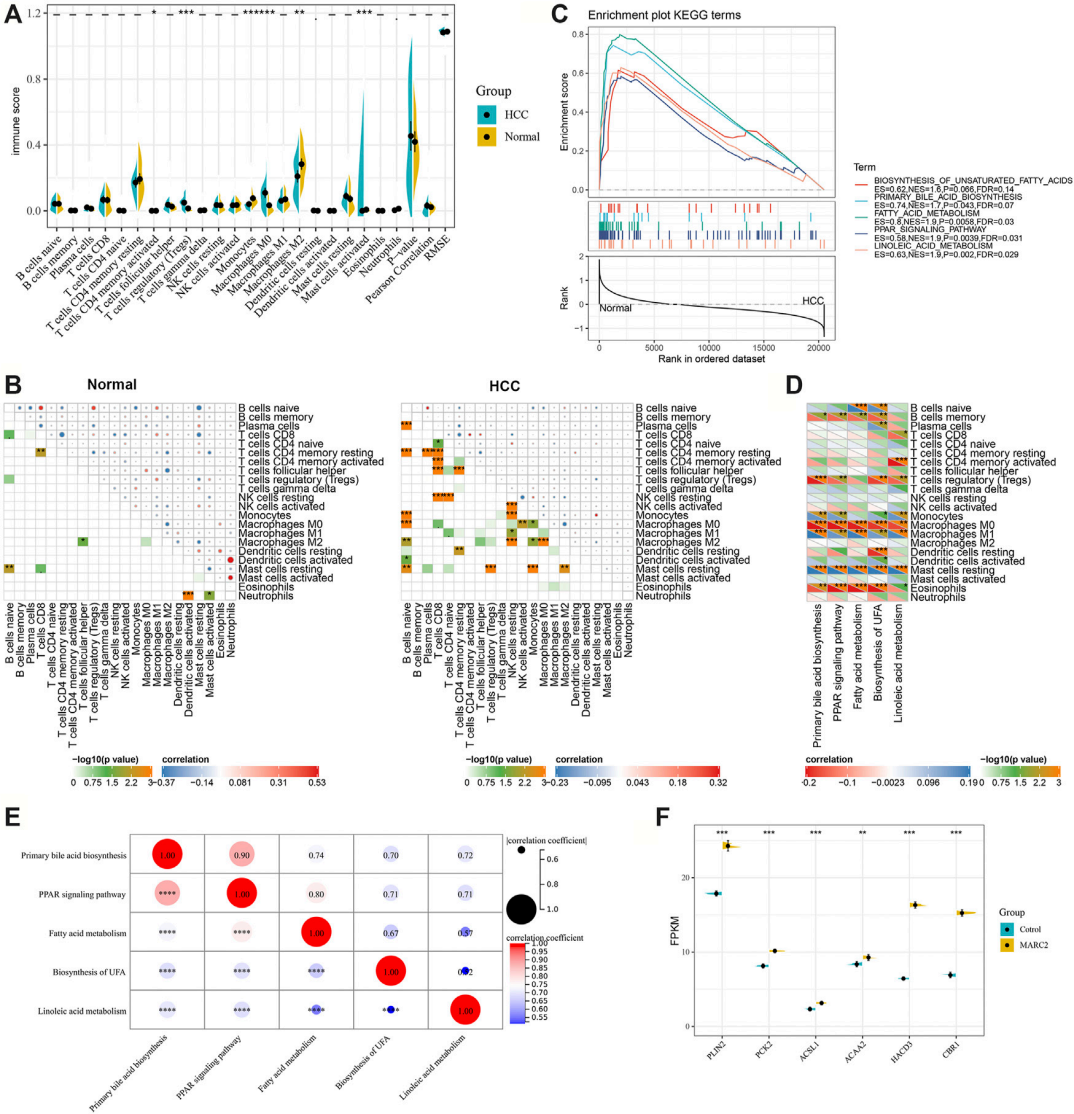
There was a correlation between MARC2-related lipid metabolism and immune modification. **(A)** Compared different immune cell types between normal and tumor tissues. **(B)** The interaction of immune cell types in normal and tumor tissues. **(C)** GSEA results revealed MARC2 related lipid metabolism signaling pathways. **(D)** The correlation between MARC2 related lipid metabolism signaling pathways and immune cell types. **(E)** The correlation between MARC2 related lipid metabolism signaling pathways. **(F)** Overexpression MARC2 in HCCLM3 cell line could regulate the expression of genes related to lipid metabolism. The Pearson correlation coefficient was used to determine the correlation. *p<0.05; **p<0.01; ***p<0.001; ***p<0.0001.

Only several immune cell types that were negatively correlated with MARC2 expression, including Tregs and M0 cells, showed significant differences between normal and tumor tissues (Figure 5A). However, the interconnecting between immune cell types was more complex in the tumor tissues than that in normal tissues (Figure 5B). MARC2-related lipid metabolism signaling pathways were predicted to have lower activity in tumor tissues than normal tissues (Figure 5C). The activities of these pathways were negatively correlated with Tregs and M0 cells, indicating that MARC2-related lipid metabolism was involved in MARC2-related immune modification (Figure 5D). In addition, the lipid metabolism signaling pathways showed a strong correlation with each other in tumor tissues (Figure 5E). Overexpression of MARC2 could also regulate lipid metabolism-related gene expression (Figure 5F). These results suggested that dysregulation of lipid metabolism might mediate MARC2-related immune modification.

### MARC2 Was Negatively Correlated With the Immune Checkpoint Expression

**FIGURE 6:**
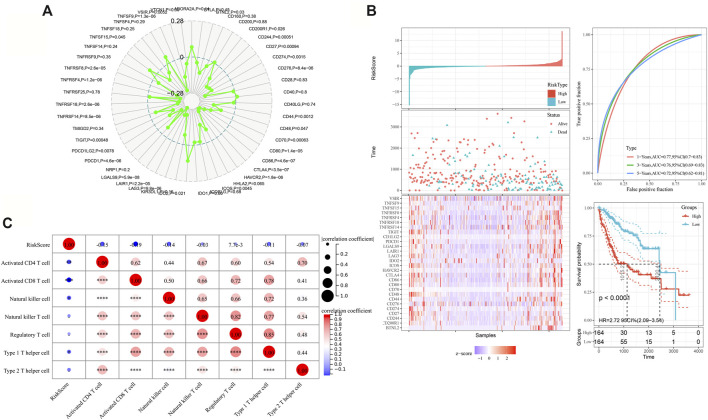
MARC2 was negatively correlated with the immune checkpoint expression. **(A)** The association between MARC2 and immune checkpoints. The immune checkpoints genes were obtained from Sangerbox. **(B)** The risk scores were created by adding up the product of coefficient of each gene and gene expression in the left panel. The ROC and Kaplan-Meier curves were used to determine the prognostic value of risk score. **(C)** The correlation between risk score and immune cell types. The Pearson correlation coefficient was used to determine the correlation. *p<0.05; **p<0.01; ***p<0.001; ***p<0.0001.

MARC2 expression had a significant negative correlation with several immune checkpoints, including the most current immune checkpoints, PDCD1 and CTLA4, and emerging focus of immunotherapy, LAG3 and TIGIT (Figure 6A). A total of 28 MARC2-related immune checkpoints (MICPs) were filtered (*p* < 0.05). The risk scores of the MICPs were generated by a stepwise multivariate Cox regression analysis, as described above, to represent the MICP burden (Figure 6B). The MICP burden was significantly correlated with survival in HCC patients, as indicated by the Kaplan-Meier curve and ROC (receiver operating characteristic) curve (Figure 6B). Patients with a higher MICP burden were predicted to have a poorer prognosis. Additionally, the MICP burden showed a moderate negative correlation with the level of activated CD8+T cells (Figure 6C). The results revealed that MARC2 staining might help select ideal candidates for immune checkpoint therapy.

## Discussion

With the success of immunotherapy in melanoma patients, the immune checkpoint inhibitors have been tested in numerous malignant tumors, including HCC (Sharma and Allison, 2015; Pinter et al., 2021). Emerging evidence confirms the safety and efficacy of anti-PD1 immunotherapy in HCC patients with advanced tumor stage (Scheiner and Pinter, 2019). TME, TMB, and MSI were the major influencing factors for immunotherapy.

In the tumor microenvironment (TME), non-malignant cells and inflammatory factors derived from stroma and tumor cells can regulate tumor progression. The immunosuppressive features of the TME are also a barrier for immunotherapy (Lu et al., 2019). Multiple immune cells coexist and interact in the context of a complex microenvironment, resulting in the failure of anti-tumor immune surveillance (Lu et al., 2019). For instance, tumor-associated macrophage polarization attracts Tregs by producing chemokines that impede the activation of cytotoxic T cells (Wang et al., 2019). Furthermore, activated cancer-associated fibroblasts by BMP4 switch NK cells to an inactive phenotype to facilitate the immune escape of tumor cells (Li et al., 2012; Mano et al., 2019). Strategies for targeting immunosuppressive cells have been actively studied. These studies provide new insights into strengthening immunotherapy.

TMB and MSI are the most definitive markers of response to immunotherapy. Neoantigens derived from somatic mutations expressed only by tumor cells are ideal targets and might enable tumor destruction by tumor-targeting lymphocytes without causing unnecessary damage to vital healthy tissues (Yamamoto et al., 2019). Thus, tumor neoantigen identification can facilitate personalized immunotherapy (Chen et al., 2019). Loss of class 1 MHC expression provides a direct mechanism of tumor immune escape (Tran et al., 2016).

The NRES is responsible for the reduction of N-oxygenated compounds and plays an important role in the activation of N-hydroxylated prodrugs and detoxification of physiological metabolites. MARC2, as a member of the NRES, is associated with tumor development. Our previous study found that decreased MARC2 promotes HCC proliferation by regulating the expression of p27 (Wu et al., 2020). In the present study, we found that NRES levels were significantly related to the progression of HCC. The risk score based on the Cox regression analysis implied that the prognostic value of NRES and MARC2 contributed significantly to predicting the prognosis of HCC. MARC2 was frequently downregulated in HCC, and restoring the expression of MARC2 could facilitate tumor antigen presentation, which is correlated with the MARC2-related lipid metabolism. The diminished expression of MARC2 was also involved in the differentiation of CD4+T cells into Tregs. The MICP burden showed important prognostic value and was negatively correlated with activated CD8+T cell levels. These results demonstrated the role of MARC2 in the tumor immune microenvironment, and the loss of MARC2 promoted immune escape and was associated with immunosuppression in HCC. MARC2 could be used as a marker for immunotherapy.

There are also several limitations in this study. Downregulate MARC2 is related with dysregulation of lipid metabolism and energy pathway. The direct mechanism of MARC2 mediated immune escape via this dysregulation has not been exposed. And whether the metabolism dysregulation induced by lacking MARC2 could drive the initiation of HCC is also need to be further investigated.

## Data Availability

The original contributions presented in the study are included in the article/Supplementary Material, further inquiries can be directed to the corresponding author.
