# Supplementary figures and images for: Downregulation of MARC2 Promotes Immune Escape and Is Associated With Immunosuppression of Hepatocellular Carcinoma
Source: Front Genet. 2022 Jan 31;12:790093. doi: 10.3389/fgene.2021.790093 (PMC8841793; doi:10.3389/fgene.2021.790093)

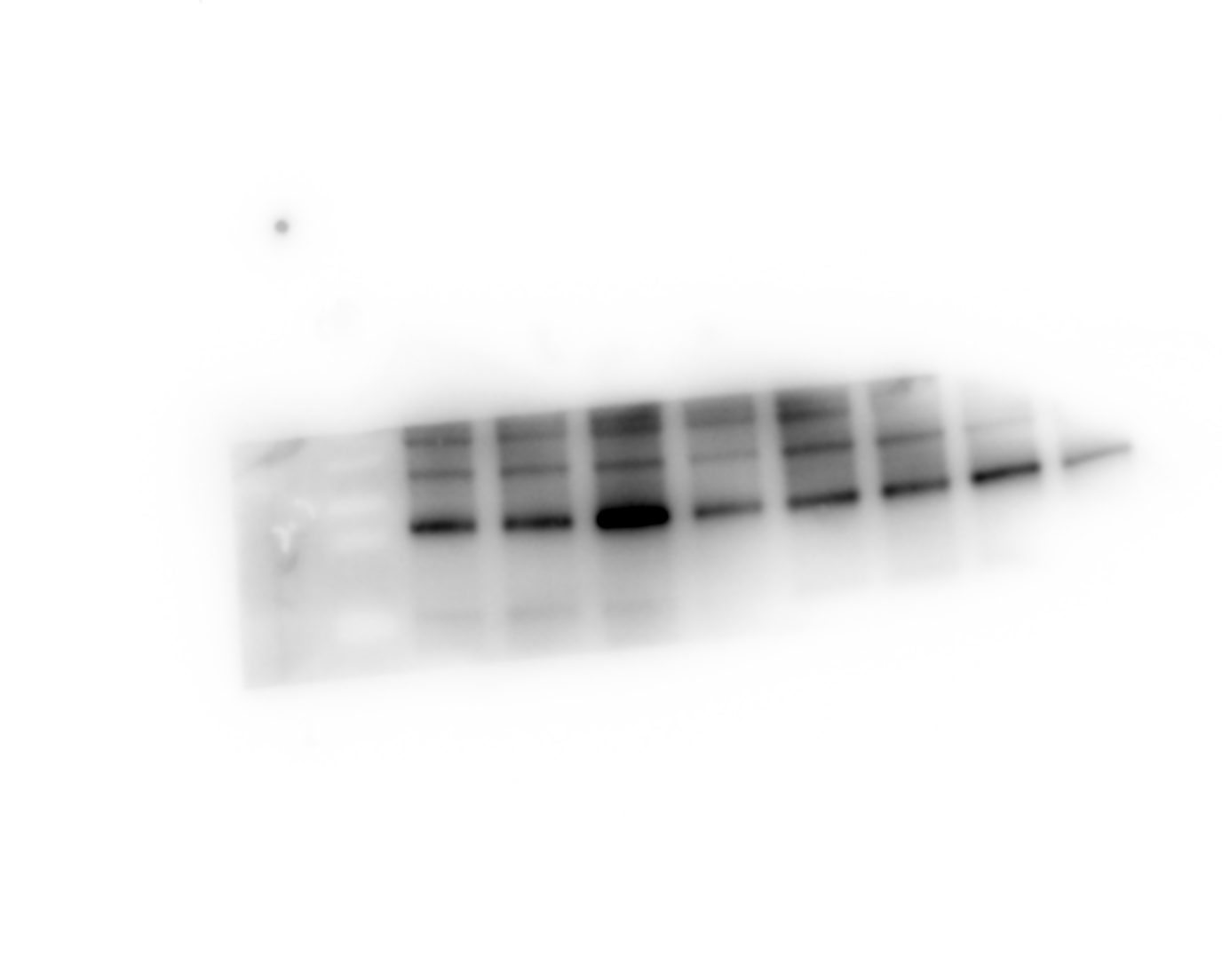

Supplement: Supplementary file 1 [file Image6.TIF]

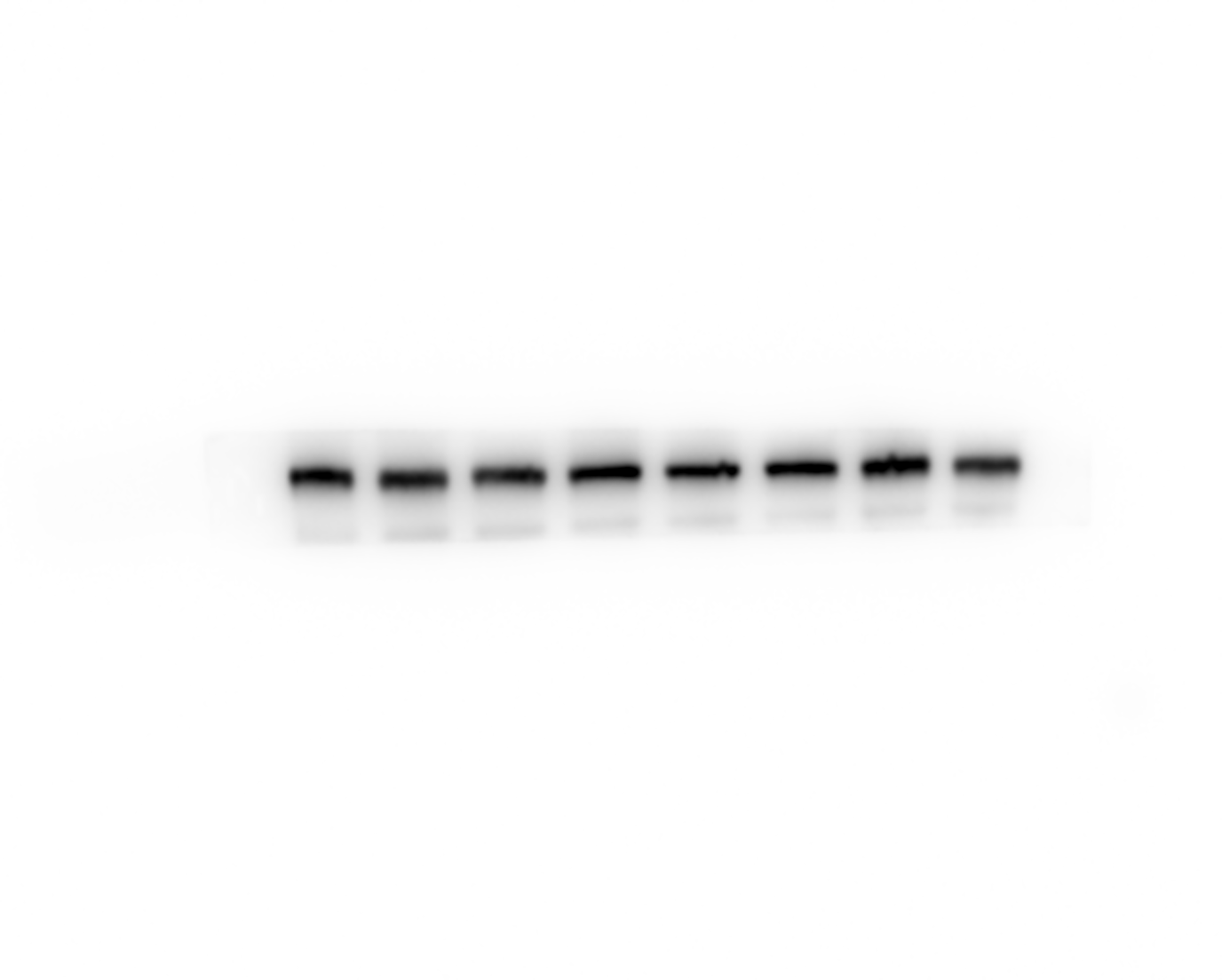

Supplement: Supplementary file 2 [file Image3.TIF]

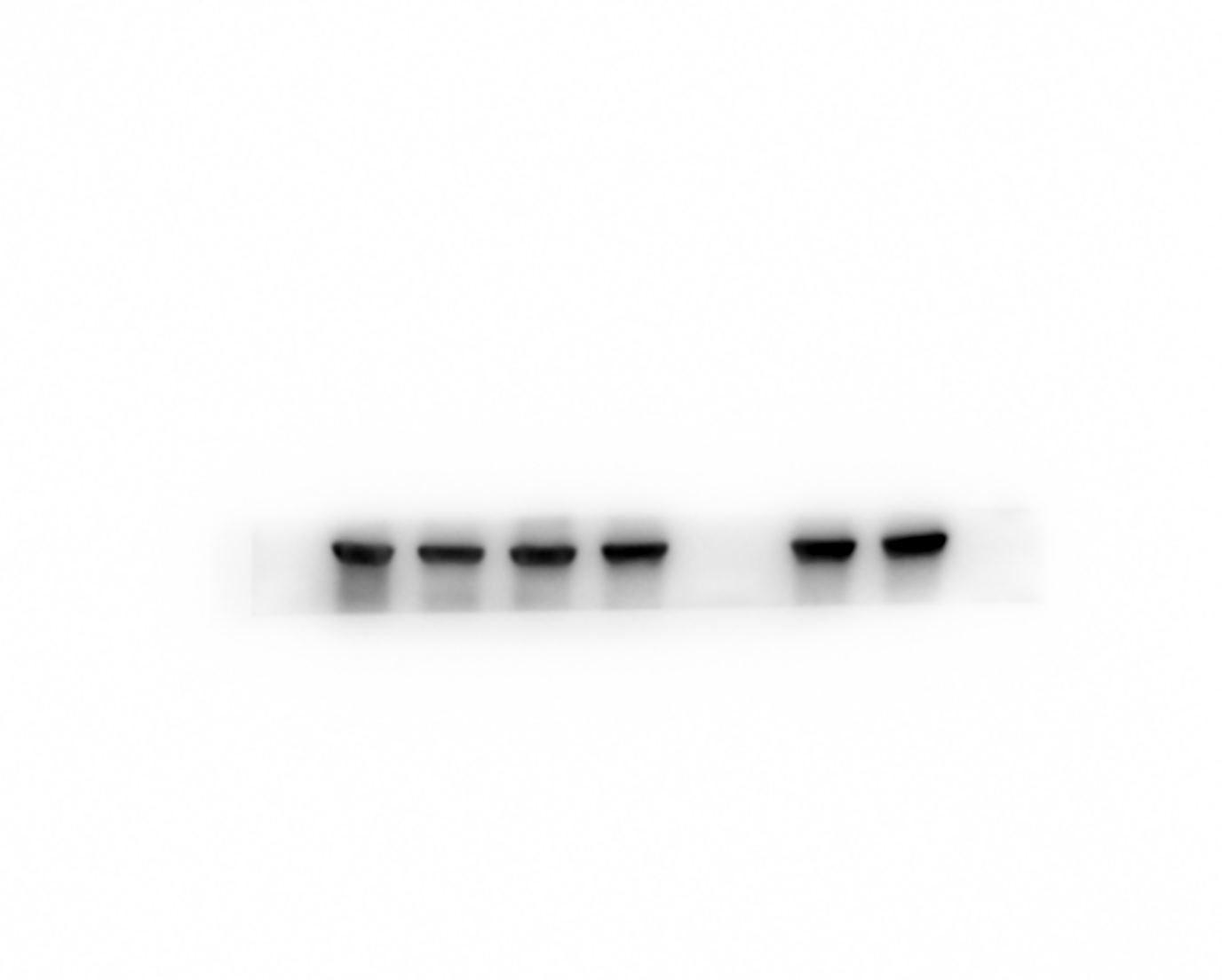

Supplement: Supplementary file 3 [file Image4.TIF]

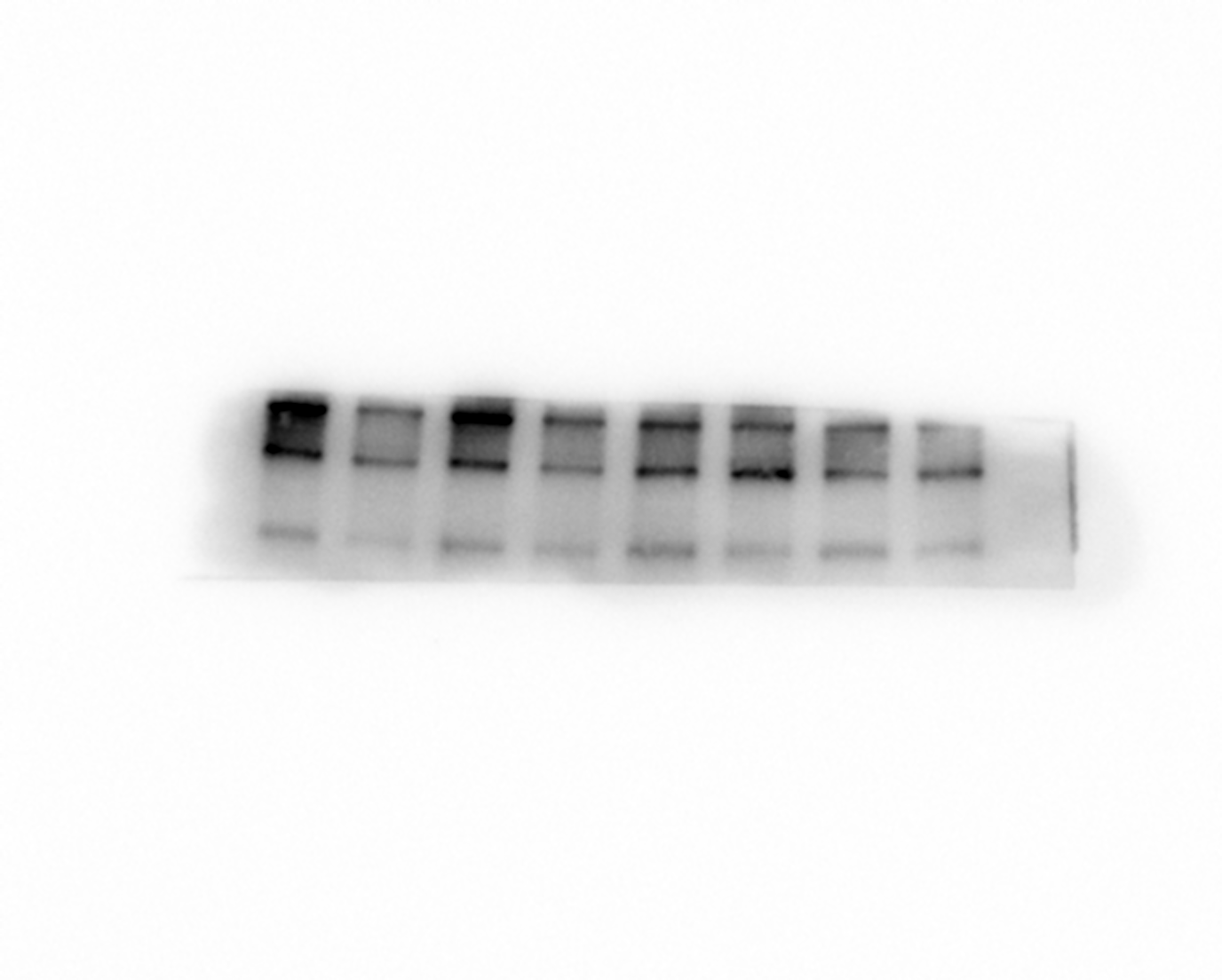

Supplement: Supplementary file 4 [file Image9.TIF]

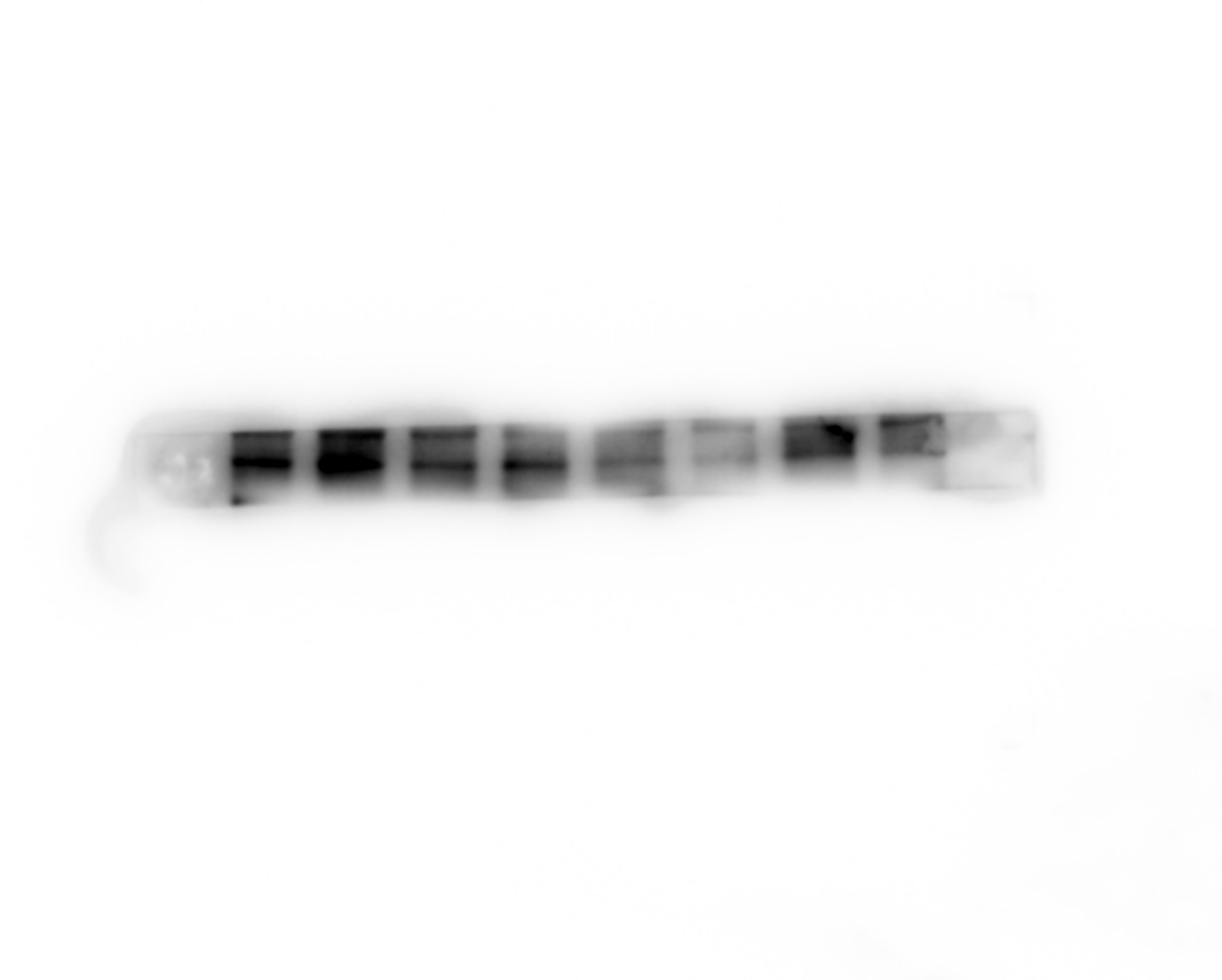

Supplement: Supplementary file 5 [file Image2.TIF]

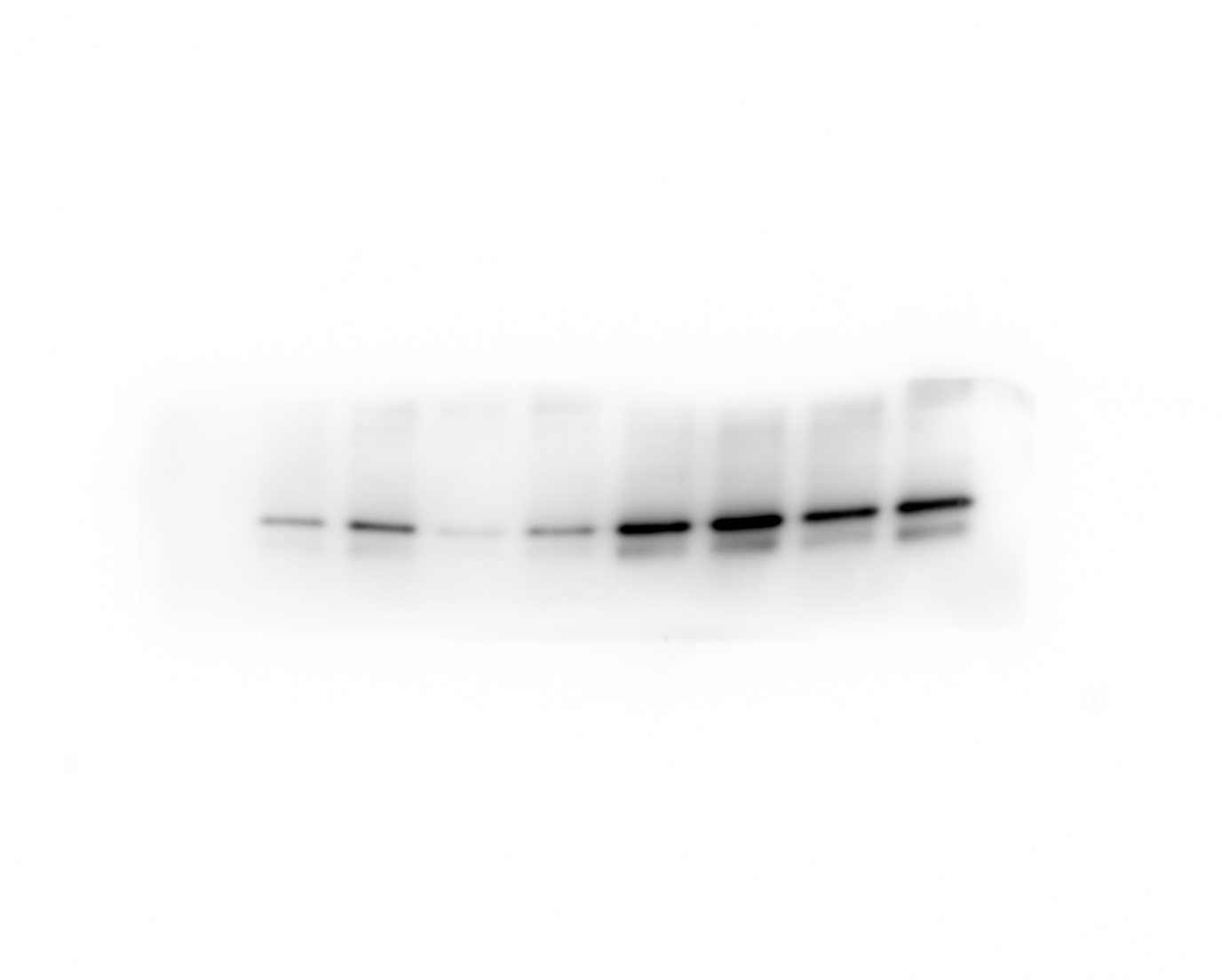

Supplement: Supplementary file 6 [file Image1.TIF]

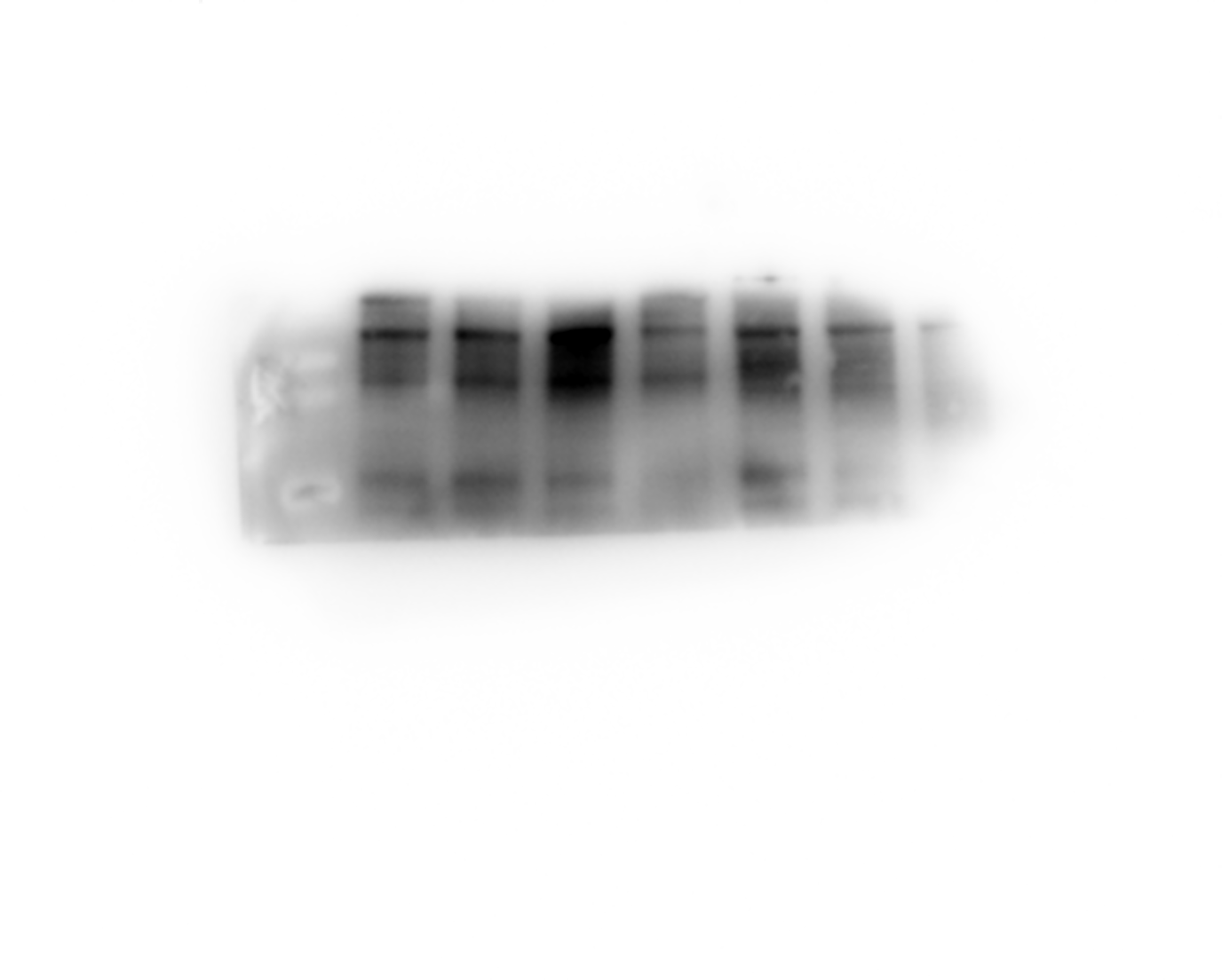

Supplement: Supplementary file 7 [file Image10.TIF]

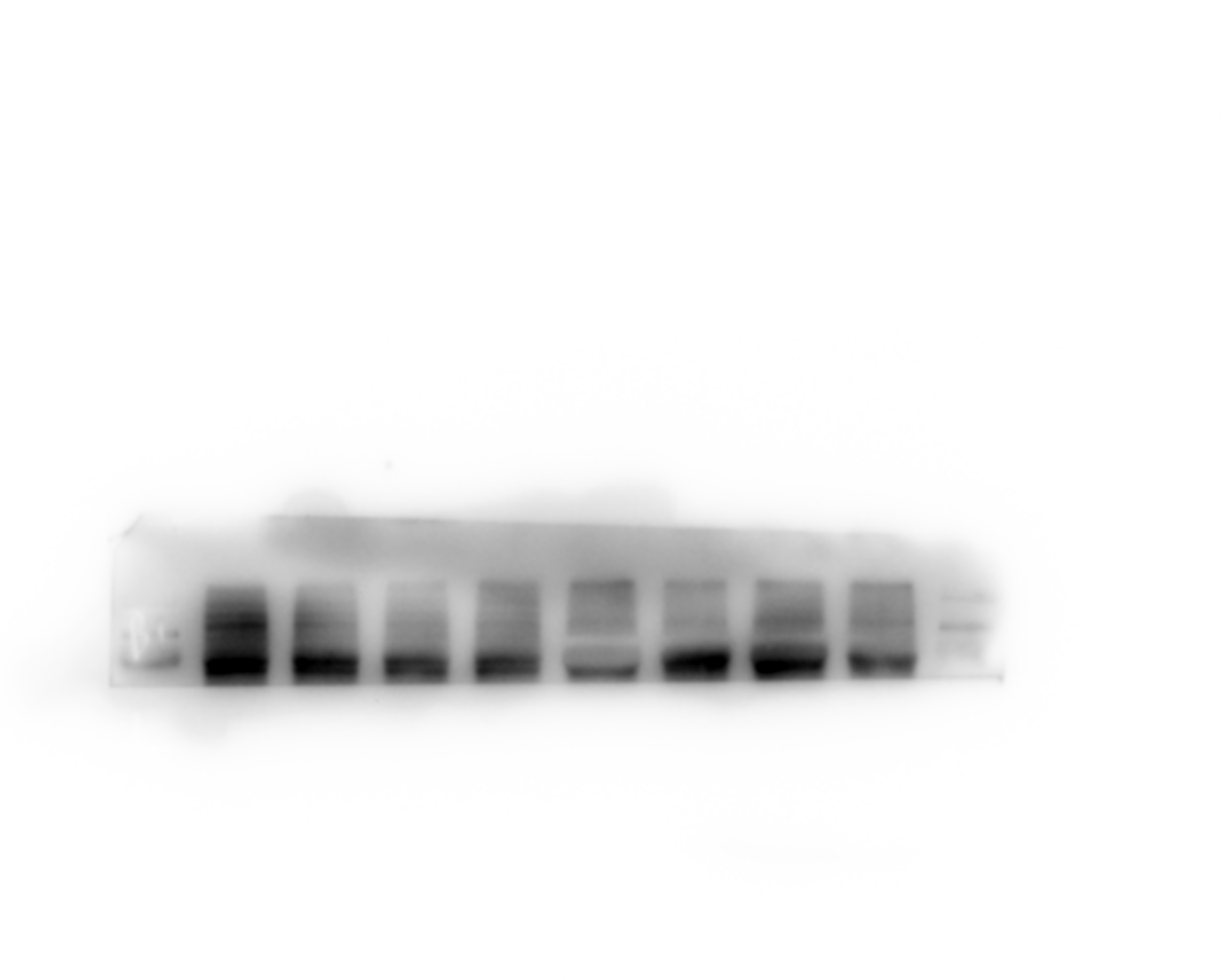

Supplement: Supplementary file 8 [file Image7.TIF]

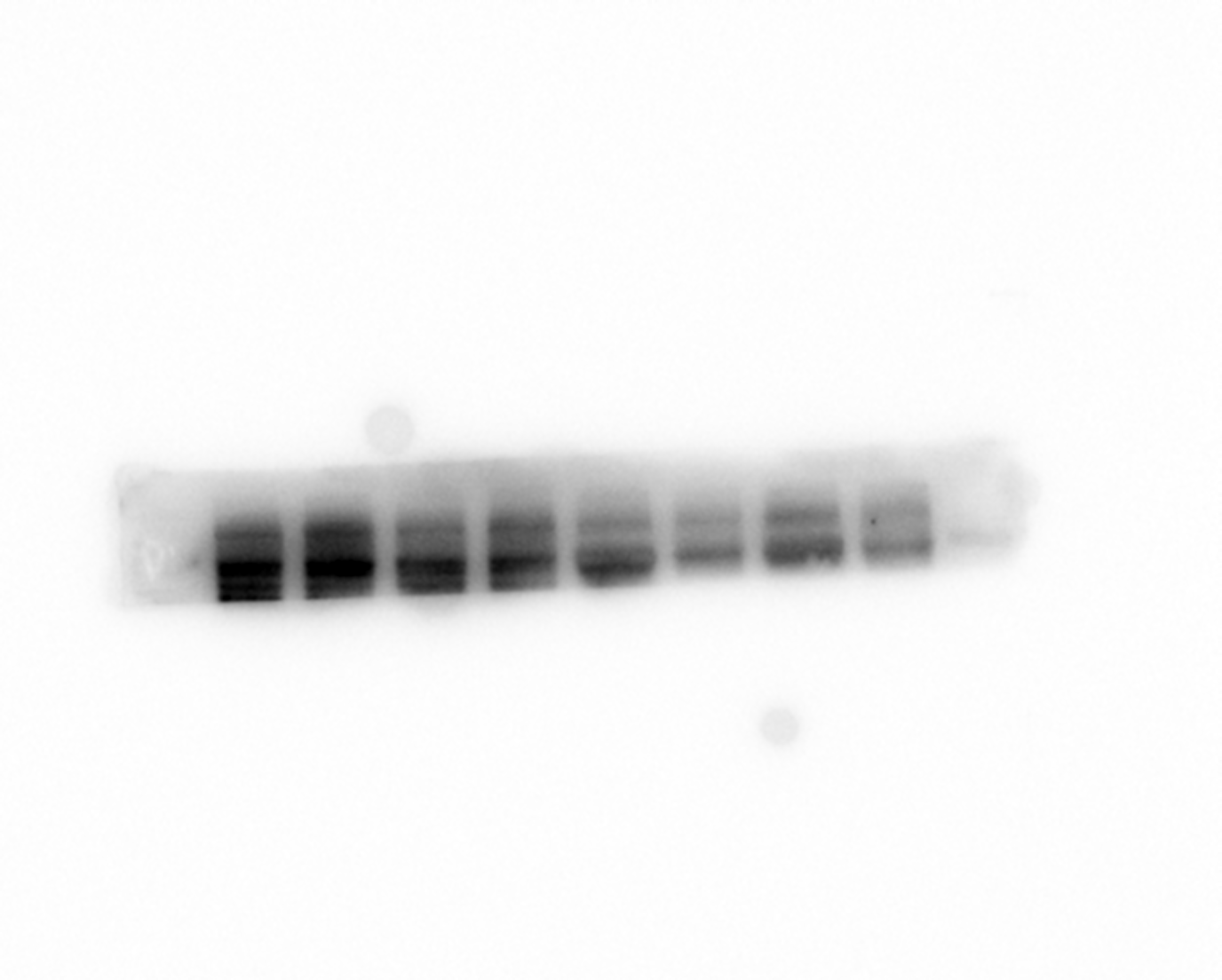

Supplement: Supplementary file 10 [file Image8.TIF]

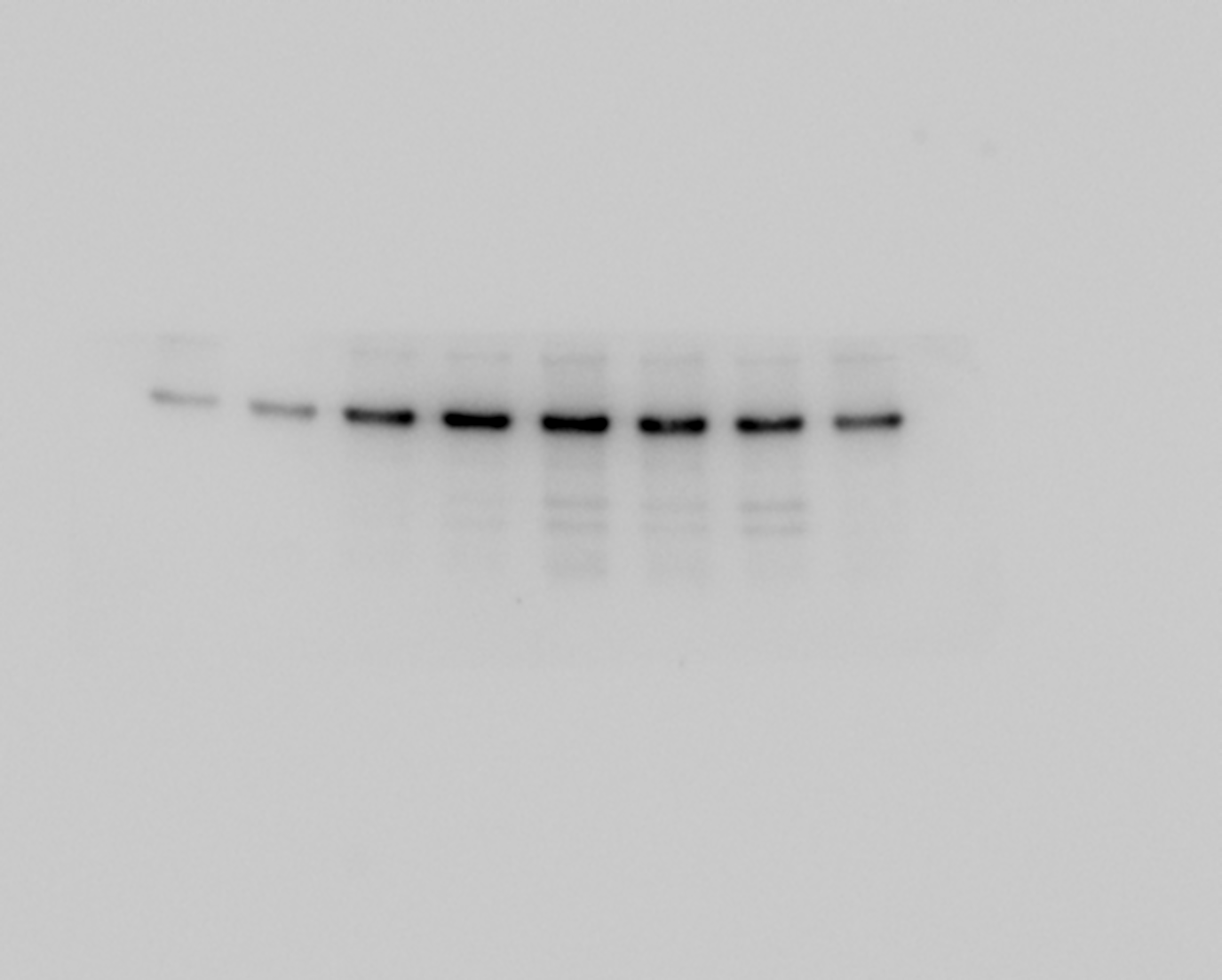

Supplement: Supplementary file 11 [file Image5.TIF]
