# Supplementary material for: Downregulation of MARC2 Promotes Immune Escape and Is Associated With Immunosuppression of Hepatocellular Carcinoma
Source: Front Genet. 2022 Jan 31;12:790093. doi: 10.3389/fgene.2021.790093 (PMC8841793; doi:10.3389/fgene.2021.790093)
Supplement: Supplementary file 12 [file DataSheet1.docx]

All the data used in this study were obtain from TCGA and GEO. There were two sources for TCGA-LIHC and GEO data Cbioportal (http://www.cbioportal.org/) and SangerBox (<http://sangerbox.com/Tool>). The data was analyzed in Sangerbox gene section and tool section. All the IMMUNE SCOREs were generated from SangerBox

Fig 1. Data derived from TCGA-LIHC and GEO14520. All the data were analyzed by SangerBox2.0 tool

Fig 2. Data derived from TCGA-LIHC and survival data was obtained from Sangerbox gene section. The data was analyzed by SangerBox2.0 tool.

Fig 3. Data derived from TCGA-LIHC. Fig 3A was analyzed by TIMER (<http://cistrome.dfci.harvard.edu/TIMER/>). Fig 3B and D were analyzed by Sangerbox 2.0 and Fig 3C was analyzed by Sangerbox3.0

Fig 4. Data derived from TCGA-LIHC and survival data was obtained from Sangerbox gene section. Fig 4B was analyzed by Sangerbox3.0 and Fig 4C-E were analyzed by Sangerbox 2.0

Fig 5. Data derived from TCGA-LIHC. Fig 5A-D were analyzed by Sangerbox 2.0 and Fig 5E was analyzed by Sangerbox3.0

Fig 6. Data derived from TCGA-LIHC. Fig 6A was obtained from Sangerbox gene section. Fig 6B was analyzed by Sangerbox 2.0 and Fig 6C was analyzed by Sangerbox3.0
